# Prevalence and risk factors for major arterial bleeding in fragility pelvic fractures in the aging population

**DOI:** 10.1038/s41598-025-22076-1

**Published:** 2025-10-31

**Authors:** Shokei Matsumoto, Makoto Aoki, Masayuki Shimizu

**Affiliations:** 1 Department of Trauma and Emergency Surgery, Saiseikai Yokohamashi Tobu Hospital, 3-6-1 Shimosueyoshi, Tsurumi-ku, Yokohama, 230-0012 Kanagawa Japan; 2 Division of Traumatology, Research Institute, National Defense Medical College, Tokorozawa, Japan

**Keywords:** Fragility pelvic fracture, Major arterial bleeding, Therapeutic angioembolization, Elderly trauma, In-hospital mortality, Diseases, Health care, Medical research, Risk factors

## Abstract

Fragility pelvic fractures (FPF) are increasingly common among elderly patients and, despite being caused by low-energy mechanisms, can result in major arterial bleeding (MAB). However, few studies have examined the prevalence and predictors of MAB in this population. This study aimed to evaluate prevalence and potential risk factors associated with MAB in elderly patients with FPF. This retrospective cohort study used data from the Japan Trauma Data Bank. The study included patients (age ≧ 65 years) with FPF between 2010 and 2021, defining FPF as pelvic fractures caused by a ground-level fall. Demographic, comorbidities, and antithrombotic use were analyzed. MAB was operationally defined by the performance of therapeutic angioembolization (TAE). Multivariable logistic regression was performed to identify predictors of MAB and in-hospital mortality. A total of 1,354 patients met inclusion criteria; 80 patients (5.9%) underwent TAE. Compared with the non-MAB group, the MAB group had significantly higher rates of liver disease, cerebral vascular disease, use of antithrombotic agents, systolic blood pressure (SBP) < 90 mmHg, and lower Glasgow Coma Scale scores. A multivariable logistic regression analysis revealed that age (OR 1.04, 95% CI 1.01–1.07), male sex (OR 2.42, 95% CI 1.49–3.93), cerebral vascular disease (OR 2.11, 95% CI 1.21–3.67), liver disease (OR 6.23, 95% CI 2.36–16.40), and SBP < 90 mmHg (OR 5.43, 95% CI 2.96–9.97) were independently associated with MAB. MAB itself was independently associated with higher in-hospital mortality (OR 4.3, 95% CI 2.1–8.9). Notably, 28% of in-hospital deaths occurred within 48 h and 50% within 7 days of admission. Despite their low-energy origin, FPFs can lead to life-threatening hemorrhage requiring invasive hemostatic intervention. A subset of elderly patients—particularly those with comorbidities or hypotension upon admission—are at elevated risk of MAB and poor outcomes. Early recognition and targeted intervention strategies are essential to improve survival in this growing and vulnerable population.

**Supplementary Information:**

The online version contains supplementary material available at 10.1038/s41598-025-22076-1.

## Introduction

 The global aging population is experiencing a rapid increase, leading to a consequential rise in the incidence of “fragility fractures” caused by low-energy mechanisms, posing a significant health concern. Several studies have documented a rising incidence of fragility pelvic fractures (FPF) in older patients. Pelvic fractures in the United States accounted for 7% of osteoporosis-related fractures and exhibited rapid growth in the disease burden^1^. A nationwide epidemiological study from Finland revealed that between 1970 and 2013, the incidence of fragility pelvic fractures among older individuals increased dramatically by 3072%^2^.

FPF are associated with underlying factors such as frailty, malnutrition, and osteoporosis, with a known poor prognosis^3^. In the treatment process for pelvic fractures in older patients, prolonged bed rest is often necessitated, which has garnered attention due to its association with complications such as pneumonia, cardiopulmonary issues, and thromboembolic events^4^. Despite the documentation of pelvic fractures with associated bleeding at the case report level, there is a conspicuous paucity of large-scale studies investigating the incidence of life-threatening bleeding events during the acute phase^5–7^. Pelvic fractures with associated hemorrhagic shock are severe conditions with poor prognoses, regardless of age.

Rapid detection and control of hemorrhage are paramount in patients with pelvic fractures presenting in hemorrhagic shock, given their high morbidity and mortality rates^8–10^. Angioembolization has demonstrated significant improvements in survival and are recommended for early bleeding control. Furthermore, other interventions such as pelvic binders, pelvic packing, external fixation (EF), and resuscitative endovascular balloon occlusion of the aorta (REBOA) in fractures resulting from low-energy trauma might be needed (Supplementary information 1). Despite the serious nature of pelvic fracture with hemorrhagic shock, such injuries are often underestimated when caused by low-energy trauma, leading to delays in recognizing and treating hemorrhagic complications. While various studies have identified risk factors for bleeding in pelvic fractures, there is a dearth of research on the specific risk factors and prevalence of FPF related major arterial bleeding (MAB) in elderly patients. This study aimed to: (1) describe the prevalence of MAB requiring therapeutic angioembolization (TAE) in elderly patients with FPF; (2) evaluate clinical outcomes (in-hospital mortality) associated with MAB, and identify independent risk factors predicting the need for TAE. We hypothesized that older age, male sex, specific comorbidities, and hemodynamic instability are associated with increased risk of MAB.

## Materials and methods

### Data source and patient selection

Data from the Japan Trauma Data Bank (JTDB) from 2010 to 2021 were retrospectively reviewed. The JTDB, established in 2003, is a nationwide trauma registry in Japan. As of 2021, more than 300 major emergency centers, defined as tertiary-level emergency facilities capable of providing advanced trauma care including surgery, angiography, and critical care, participated in the registry. The JTDB enrolls patients primarily with injuries classified as Abbreviated Injury Scale (AIS) ≥ 3, although institutions can voluntarily register all trauma patients regardless of severity. Ethical approval for this study was granted by the Japanese Association for the Surgery of Trauma and the institutional review boards of all participating hospitals. As only anonymized data from the JTDB were used, the requirement for informed consent was waived by the Ethics Committee of the Japanese Association for the Surgery of Trauma and the National Defense Medical College (approval ID No. 2019-02-迅速−02), in accordance with the Ethical Guidelines for Medical and Health Research Involving Human Subjects of the Ministry of Health, Labour and Welfare of Japan. The approval document is publicly available on the JTDB website (https://www.jtcr-jatec.org/traumabank/dataroom/ethics2.htm). This study also complied with the STROBE guidelines and was conducted in accordance with the 1964 Helsinki declaration and its later amendments.

The study included elderly patients aged ≥ 65 years with FPF, defined as pelvic fractures resulting from ground-level falls. Pelvic fractures were identified according to the AIS codes (see Supplementary information 2).

Patients were excluded if they sustained injuries from non-ground-level falls, were transferred from a referring institution, were dead on arrival, or had an AIS score of 6 in any other body region.

## Outcomes and data collection

Demographic variables included age, sex, vital signs on arrival, Injury Severity Score (ISS), Glasgow Coma Scale (GCS), cause of injury, concomitant injuries (AIS of the head, chest, and abdomen), and pelvic computed tomography (CT) scans. Emergency procedures for pelvic fractures (angiography, angioembolization, EF, and REBOA) were identified from the relevant sections of the JTDB records. MAB was defined as cases where angioembolization was performed to control pelvic hemorrhage. The primary outcome was the prevalence of MAB and its impact on survival outcomes. We also aimed to identify predictors of MAB in elderly patients with FPF.

### Statistical analysis

Descriptive statistics were calculated to summarize the data. Patients were stratified into two groups: those with MAB (MAB group) and those without (non-MAB group). Continuous variables are presented as mean ± standard deviation (SD) or median with interquartile range (IQR), depending on distribution, and categorical variables are expressed as frequencies and percentages. Normality of continuous variables was assessed using the Shapiro–Wilk test.

Between-group comparisons were performed using the Student’s t-test or the Mann–Whitney U test for continuous variables, and the Pearson’s chi-squared test or Fisher’s exact test for categorical variables, as appropriate.

To identify independent predictors of MAB and in-hospital mortality, separate multivariable logistic regression models were constructed. Candidate variables included clinically relevant factors and those with a p-value < 0.10 in univariate analysis. A stepwise selection method was then applied, retaining variables with p-values < 0.05 in the final model. Results are reported as odds ratios (ORs) with 95% confidence intervals (CIs), and statistical significance was defined as a two-tailed p-value of < 0.05.

Missing data were addressed using multiple imputation with chained equations (MICE), employing predictive mean matching across 10 iterations to ensure convergence. All statistical analyses were conducted using Python (version 3.11.8).

In addition, two sensitivity analyses were conducted: one excluding patients with abdominal AIS ≥ 3, to address the limitation that TAE may have been performed for intra-abdominal rather than pelvic bleeding; and another excluding patients with head, chest, or abdominal AIS ≥ 3, to account for the uncertainty of causes of death in patients with severe extra-pelvic trauma.

## Results

### Patients characteristics

**Fig. 1:**
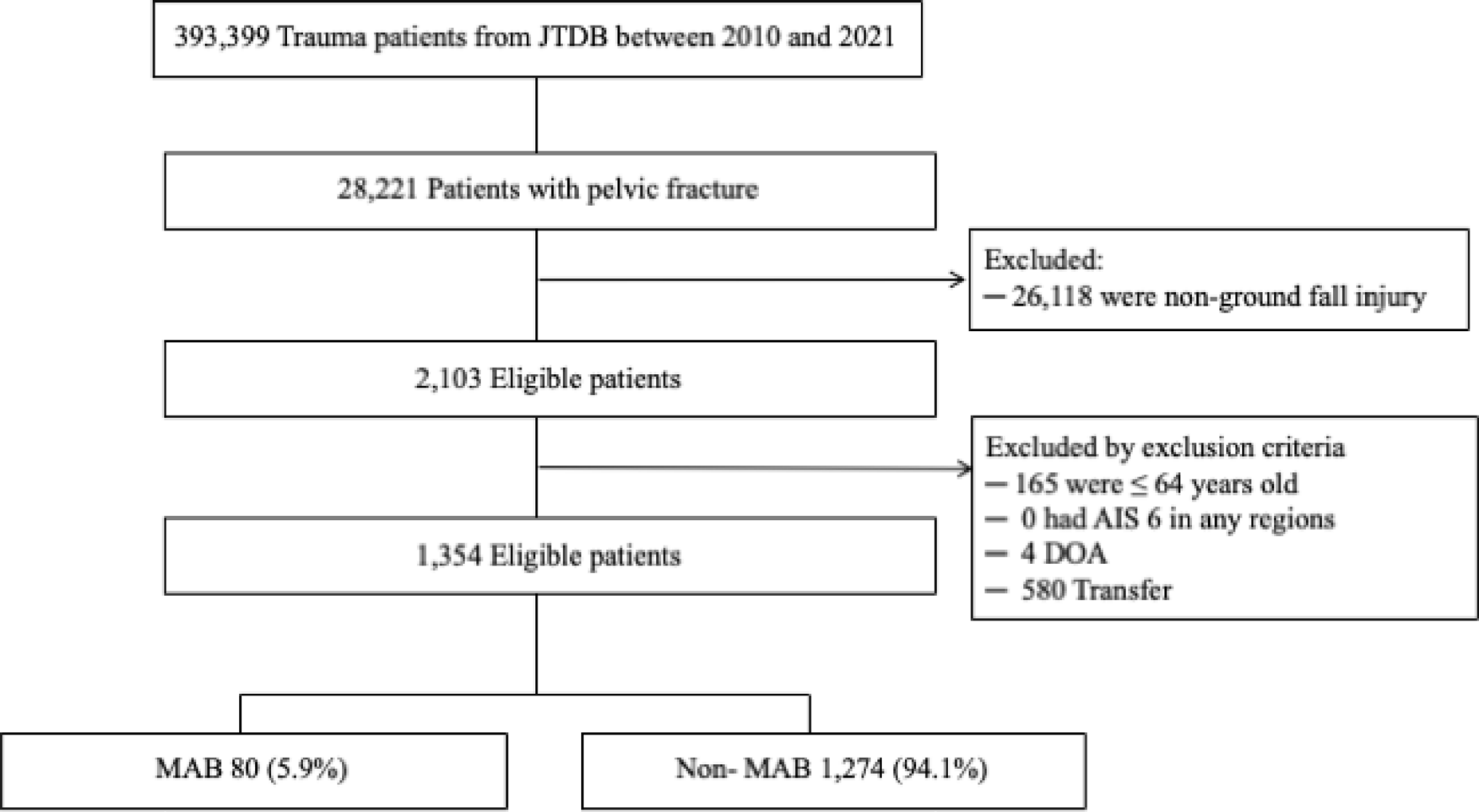
Patient selection flowchart from the Japan Trauma Data Bank (JTDB), 2010–2021. MAB, major arterial bleeding requiring therapeutic angioembolization; DOA, dead on arrival.

A total of 1,354 patients met the inclusion criteria, among whom 80 (5.9%) underwent angioembolization. The patient selection flowchart is shown in Fig. 1. Baseline clinical characteristics are summarized in Table 1. The majority of patients were female (74.4%), and the median age was 85.0 years (IQR 79.0–90.0). The median systolic blood pressure (SBP) at admission was 141 mmHg (IQR 120–161), and the median Injury Severity Score (ISS) was 9.0 (IQR 4.0–13.0).

**Table 1:** Baseline characteristics of elderly patients with pelvic fracture due to Ground-Level Falls, stratified by presence of major arterial Bleeding.

Demographic Characteristics	Non-MAB (*n* = 1274)	MAB (*n* = 80)	Total (*n* = 1354)	*P*-value
Male sex, n (%)	314 (24.6)	34 (42.5)	348 (25.6)	0.001
Age, median	85 (79–89)	86 (82–90)	85 (79–90)	0.098
Age group (y), n (%)				
65–69	71 (5.6)	1 (1.2)	72 (5.3)	0.166
70–74	117 (9.2)	5 (6.2)	122 (9.0)	
75–79	156 (12.2)	7 (8.8)	163 (12.0)	
80–84	246 (19.3)	16 (20.0)	262 (19.3)	
85–89	365 (28.6)	28 (35.0)	393 (29.0)	
90–94	223 (17.5)	12 (15.0)	235 (17.3)	
95+	96 (7.5)	11 (13.8)	107 (7.9)	
Vital signs on hospital arrival				
SBP, mm Hg	142 (122–162)	117 (94–144)	141 (120–161)	< 0.001
SBP < 90 mmHg, n (%)	63 (4.9)	18 (22.5)	81 (5.9)	< 0.001
HR, bpm	83 (72–94)	81 (71–95)	82 (72–95)	0.879
HR > 120 bpm, n (%)	38 (3.0)	5 (6.2)	43 (3.1)	0.105
Temperature, °C	36.7 (36.3–37.1)	36.3 (35.9–36.6)	36.7 (36.3–37.1)	< 0.001
GCS score	15 (14–15)	15 (13–15)	15 (14–15)	< 0.001
GCS < 9, n (%)	35 (2.7)	8 (10.0)	43 (3.1)	0.003
ISS	9 (4–13)	16 (14–25)	9 (4–13)	< 0.001
Severe injured region, AIS score				
Head AIS ≥ 3, n (%)	92 (7.2)	6 (7.5)	98 (7.2)	0.826
Chest AIS ≥ 3, n (%)	89 (7.0)	8 (10.0)	97 (7.2)	0.366
Abdomen AIS ≥ 3, n (%)	14 (1.1)	10 (12.5)	24 (1.8)	< 0.001
Upper extremity AIS ≥ 2, n (%)	148 (11.6)	5 (6.3)	153 (11.3)	0.200
Lower extremity AIS ≥ 3, n (%)	531 (41.7)	62 (77.5)	593 (43.8)	< 0.001
Femur fracture, n (%)	122 (9.6)	5 (6.2)	127 (9.3)	0.429

Continuous variables were presented as the mean ± SD or the median (IQR). Categorical variables were presented as number (%).

SBP: Systolic Blood Pressure, HR: Heart Rate, GCS: Glasgow Coma Scale, ISS: Injury Severity Score, AIS: Abbreviated Injury Scale.

Regarding concomitant injuries, 7.2% had a head AIS score ≥ 3, and 1.7% had an upper extremity AIS score ≥ 2. Compared with the non-MAB group, the MAB group had a significantly higher proportion of male patients, lower SBP, lower body temperature, and lower GCS scores (all *p* < 0.05).

### Pre-existing medical condition

**Fig. 2:**
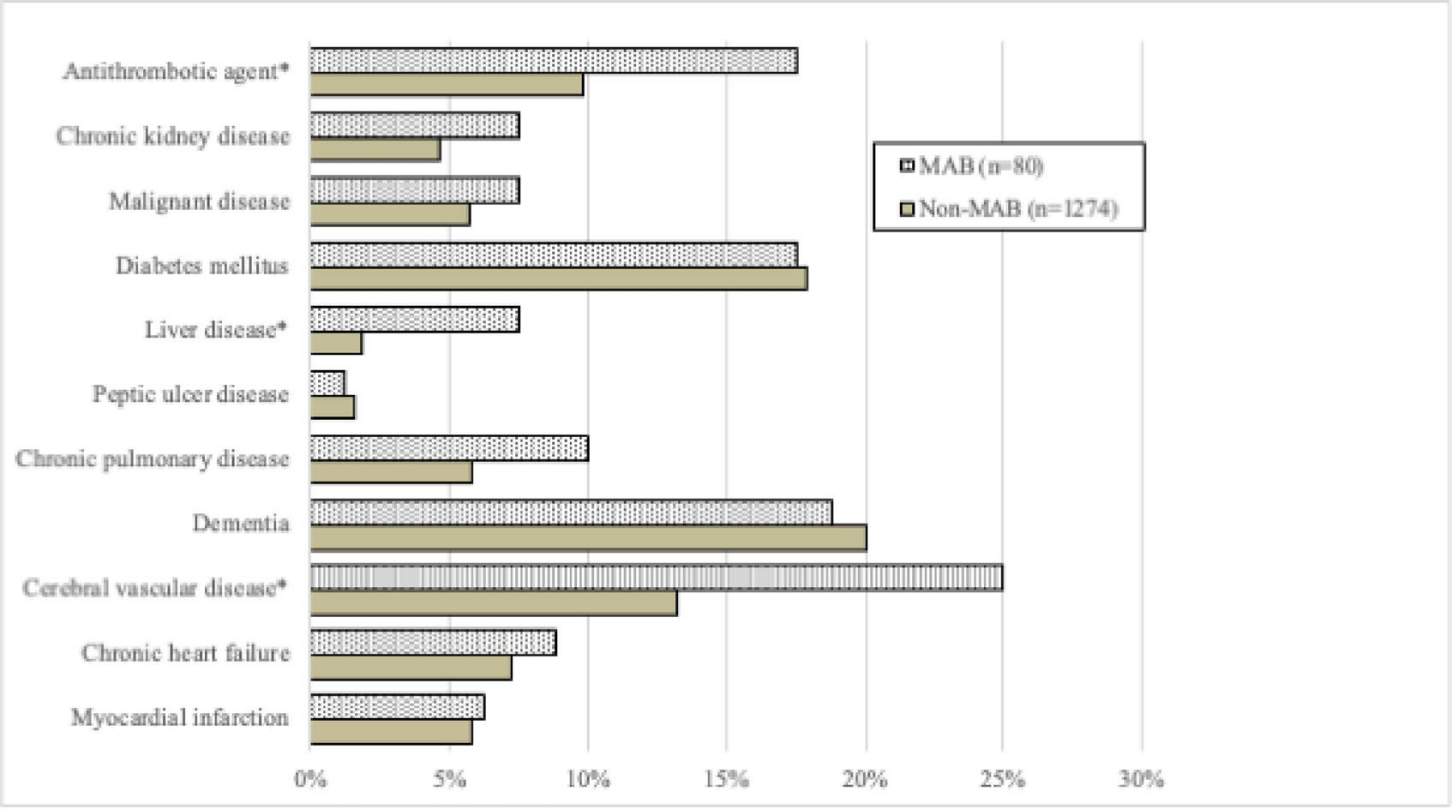
Incidence of major arterial bleeding in elderly patients with fragility pelvic fractures. The bar graph shows the proportion of patients with major arterial bleeding requiring therapeutic angioembolization across subgroups. *P < 0.05 indicates statistically significant difference.

Overall, the most common comorbidities were dementia (19.9%), diabetes mellitus (17.8%), and cerebral vascular disease (13.8%). The rates of pre-existing conditions, stratified by non-MAB and MAB groups, are shown in Fig. 2. Compared with the non-MAB group, the MAB group had significantly higher rates of antithrombotic agent use, liver disease, and cerebral vascular disease (all *p* < 0.05).

## Diagnostic imaging, treatment and outcome

Table 2 summarizes diagnostic imaging, interventions, and outcomes. Most patients underwent computed tomography (CT) scans (85.8%), while angiography was performed in a smaller subset (8.4%). Regarding therapeutic interventions, angioembolization, EF, and REBOA were performed in 80 (5.9%), 29 (2.1%), and 2 (0.1%) patients, respectively. Among patients who underwent angiography, 70.1% received TAE. The overall in-hospital mortality was 3.9%. Compared with the non-MAB group, the MAB group had significantly higher rates of blood transfusion within 24 h (11.5% vs. 62.5%, *p* < 0.001) and in-hospital mortality (3.2% vs. 16.2%, *p* < 0.001).

**Table 2:** Diagnosis, Intervention, and outcomes of elderly patients with pelvic fracture due to Ground-Level Falls, stratified by presence of major arterial Bleeding.

	Non-MAB (*n* = 1274)	MAB (*n* = 80)	Total (*n* = 1354)	*P*-value
Radiology study				
CT scan, n (%)	1,089 (85.5)	74 (92.5)	1,163 (85.8)	0.096
Angiography, n (%)	34 (2.7)	80 (100.0)	114 (8.4)	< 0.001
Blood transfusion within 24 h injury, n (%)	147 (11.5)	50 (62.5)	197 (14.5)	< 0.001
Hemostatic Intervention				
Therapeutic angioembolization, n (%)	0 (0)	80 (100.0)	80 (5.9)	< 0.001
REBOA, n (%)	2 (0.2)	0 (0)	2 (0.1)	1.000
External fixation, n (%)	22 (1.7)	7 (8.8)	29 (2.1)	0.001
ORIF, n (%)	158 (12.4)	10 (12.5)	168 (12.4)	1.000
Hospital LOS, d	19 (10–31)	24 (11–34)	19 (10–32)	0.079
Mortality, n (%)	41 (3.2)	13 (16.2)	54 (3.9)	< 0.001

Continuous variables are presented as median (interquartile range); categorical variables as number (%). CT: Computed Tomography, REBOA: Resuscitative Endovascular Balloon Occlusion of the Aorta, ORIF: Open Reduction and Internal Fixation, LOS: Length of Stay.

## Factors associated with increased bleeding requiring intervention and In-hospital mortality

A multivariable logistic regression analysis revealed that age (OR 1.04, 95% CI 1.01–1.07), male sex (OR 2.42, 95% CI 1.49–3.93), cerebral vascular disease (OR 2.11, 95% CI 1.21–3.67), liver disease (OR 6.23, 95% CI 2.36–16.40), and SBP < 90 mmHg (OR 5.43, 95% CI 2.96–9.97) were independently associated with MAB (Table 3).

**Table 3:** Multivariable logistic regression identifying predictors of major arterial bleeding in elderly patients with fragility pelvic Fractures.

Major arterial bleeding	Odds ratio	95% CI		*P*-value
Age	1.04	1.01	1.07	0.014
Male	2.42	1.49	3.93	< 0.001
Cerebral vascular disease	2.11	1.21	3.67	0.009
Liver disease	6.23	2.36	16.40	< 0.001
SBP < 90 mmHg	5.43	2.96	9.97	< 0.001
Mortality				
Male	1.98	1.11	3.54	< 0.001
GCS < 9	9.21	4.26	19.90	< 0.001
MAB	4.32	2.10	8.88	< 0.001

OR: Odds Ratio, CI: Confidence Interval, SBP: Systolic Blood Pressure, GCS: Glasgow Coma Scale, MAB: Major Arterial Bleeding.

Regarding in-hospital mortality, independent predictors included male sex (OR 1.98, 95% CI 1.11–3.54), GCS < 9 (OR 9.21, 95% CI 4.26–19.90), and the presence of MAB (OR 4.32, 95% CI 2.10–8.88) (Table 3).

To address potential confounding from associated injuries, we performed two sensitivity analyses. First, excluding patients with abdominal AIS ≥ 3, the results remained consistent with the primary analysis: male sex, hypotension (SBP < 90 mmHg), liver disease, and cerebrovascular disease were still independently associated with MAB (see Supplementary information 3 and 4). Second, excluding patients with severe head, chest, or abdominal injuries (AIS ≥ 3), the results again confirmed the robustness of the primary findings (see Supplementary information 5 and 6).

Among the 54 patients who died, 28% died within 48 h, and 50% died within 7 days of admission (see Supplementary information 7).

## Discussion

FPF represent a growing concern in elderly patients, often resulting in serious complications and increased mortality. In this nationwide study using JTDB data collected over an 11-year period, 5.9% of elderly patients with FPF developed MAB requiring TAE. To our knowledge, this is the largest multi-institutional analysis of FPF to date, providing a unique opportunity to explore risk factors associated with poor outcomes. These findings highlight the urgent need for early risk stratification and timely hemostatic intervention to prevent life-threatening hemorrhage in this vulnerable patient population.

While previous studies have identified several risk factors for FPF—such as osteoporosis, diabetes, vitamin D deficiency, hypocalcemia, and nicotine use—which contribute to higher mortality and loss of functional independence^11^, there remains a paucity of research specifically addressing the risk of MAB in FPF. A recent single-center cohort study found that clinically significant bleeding occurred in less than 1% of elderly patients with low-energy FPF, and no invasive interventions were required^12^. However, case reports and small series have described life-threatening hemorrhage in anticoagulated elderly patients with seemingly minor pelvic fractures, highlighting the importance of maintaining high clinical suspicion in select populations^6^. Moreover, a literature review found that most evidence on hemorrhage in FPF comes from isolated case reports, and comprehensive investigations into the risk factors for arterial bleeding remain scarce^5^. These findings suggest that while bleeding is uncommon overall in FPF, certain patients—particularly those with comorbidities or on antithrombotic therapy—may be at increased risk and should be carefully monitored. In contrast, our study observed a higher incidence of MAB (5.9%), likely due to the nature of the JTDB, which predominantly includes patients with moderate to severe trauma. As such, patients with mild pelvic injuries are underrepresented, potentially leading to an overestimation of bleeding incidence. Therefore, further research is warranted to better elucidate predictors of hemorrhage and to inform individualized, risk-based management strategies.

Although several management algorithms for pelvic trauma have been proposed, few are specifically tailored to elderly patients with FPF. This is particularly concerning, as elderly individuals exhibit unique physiological vulnerabilities, including decreased bone density, multiple comorbidities, and an increased risk of hemorrhage due to the frequent use of anticoagulant or antiplatelet medications^13^. Furthermore, the low-energy mechanisms commonly responsible for FPF—such as ground-level falls—are often underestimated in terms of clinical severity, leading to delayed diagnosis and intervention^12^. The lack of dedicated, evidence-based guidelines for this demographic may contribute to substantial variability in clinical decision-making. Therefore, there is an urgent need to establish standardized protocols addressing the unique challenges associated with geriatric FPF, with particular emphasis on early detection of MAB and the appropriate use of minimally invasive interventions such as TAE.

Given the rising incidence of FPF driven by global population aging, it is increasingly important to implement patient-centered strategies that address both the fracture itself and the broader complexities of geriatric trauma care. Pre-existing conditions have been associated with significantly worse outcomes in patients with fragility fractures, contributing to impaired functional recovery and higher mortality^14^. In our study, both liver disease and cerebral vascular disease were independently associated with MAB in elderly patients with FPF, underscoring the need for individualized risk stratification. These comorbidities may increase physiological vulnerability, delay recovery, and complicate hemorrhage management. Thus, future research should prioritize the refinement of treatment algorithms or the development of new, evidence-based guidelines that reflect the multifactorial nature of fragility fractures in older adults and aim to reduce both mortality and morbidity in this high-risk population.

However, it is important to note that the JTDB does not define “liver disease” with specific diagnostic criteria. The classification likely encompasses a heterogeneous range of hepatic conditions, including cirrhosis, chronic hepatitis, or even mild liver enzyme abnormalities, depending on each hospital’s reporting practices. Despite this heterogeneity, the strong association observed in our multivariate analysis (OR 6.23) suggests that underlying liver dysfunction may play a significant role in bleeding risk. Potential mechanisms may include impaired coagulation, portal hypertension, and reduced hepatic reserve, all of which may impair hemostasis or complicate resuscitation. Further research incorporating more granular clinical and laboratory data is needed to clarify these associations.

Interestingly, the incidence of severe abdominal injury was significantly higher in patients with MAB (12.5%) compared to those without (1.1%). This association suggests that abdominal trauma may serve as a contributing factor to—or a marker of—severe hemorrhagic events in the context of FPF. One possible explanation is that concurrent abdominal organ injury may exacerbate overall hemorrhage volume or complicate hemodynamic stabilization, thereby increasing the necessity for TAE. Alternatively, this finding may simply reflect that patients in the MAB group sustained higher-energy trauma and had greater overall injury severity, as evidenced by their significantly higher AIS scores for both the abdomen and lower extremities. Thus, caution is warranted when interpreting the causal relationship between associated injuries and the need for invasive hemostatic intervention. To address this potential confounding, we conducted a sensitivity analysis excluding patients with abdominal AIS ≥ 3. The results remained consistent, supporting the robustness of our primary findings. Nevertheless, the indication for TAE (i.e., pelvic vs. abdominal bleeding) was not specifically recorded in our dataset, which remains an important limitation. Future studies should aim to disentangle these overlapping factors.

Our findings highlight that early mortality is a major concern in patients with fragility pelvic fractures. Notably, more than one-quarter (28%) of in-hospital deaths occurred within the first 48 h, and cumulative mortality reached 50% by day 7. These results emphasize the urgent need for prompt recognition and early intervention, particularly during the hyperacute and subacute phases, when hemostatic control and hemodynamic stabilization may still positively influence clinical outcomes. This pattern is consistent with prior evidence indicating that most deaths within the first 6 h after pelvic fracture are due to hemorrhage, whereas deaths beyond 24 h are more often related to sepsis or multiple organ dysfunction syndrome^15^. In our study, delayed mortality beyond 7 days—accounting for the remaining 50%—may be attributable to complications such as prolonged immobility, nosocomial infections, or exacerbation of pre-existing comorbidities.

In interpreting mortality, it should be noted that the JTDB does not record precise causes of death. While early mortality in patients with FPF and MAB is most likely attributable to hemorrhage, late deaths could have been influenced by concomitant head, chest, or abdominal injuries. To address this, we performed an additional sensitivity analysis excluding patients with severe extra-pelvic trauma (AIS ≥ 3 in the head, chest, or abdomen), and the results remained consistent with the primary analysis. These findings support the robustness of our conclusions despite the absence of detailed cause-of-death information.

This study has several limitations. First, although the JTDB is a nationwide trauma registry, it is based on a convenience sample of voluntarily participating hospitals, which may introduce selection bias and limit the generalizability of the findings. However, as most major emergency centers in Japan participate in the registry, the data likely reflect national trauma care trends. Second, as a retrospective registry-based study, the analysis is susceptible to incomplete or inconsistent data entry and variations in institutional protocols, which may affect the accuracy and uniformity of clinical information. Third, although this study focused on FPFs due to ground-level falls, other low-energy mechanisms may also contribute to FPF but were not included. This may underestimate the true incidence of FPFs and limit the comprehensiveness of our analysis. Fourth, the JTDB lacks detailed anatomical classification of pelvic fracture types and does not capture specific bleeding sources or procedural details of angioembolization. In particular, the registry does not distinguish between therapeutic, prophylactic, or diagnostic angiographic procedures. Additionally, it does not specify whether angioembolization was performed for pelvic arterial hemorrhage or concomitant intra-abdominal organ injury. This lack of granularity may limit the precision in determining the true incidence and etiology of MAB. Similarly, although REBOA and EF were used in some non-MAB patients, but their indications were unclear. These procedures may have been used for non-pelvic bleeding or for stabilization rather than hemorrhage. Finally, unmeasured confounding factors, such as frailty, osteoporosis severity, or timing of interventions, may have influenced both bleeding risk and mortality, but could not be accounted for due to database limitations. Despite these limitations, this study offers valuable insights into the characteristics and clinical trajectory of elderly patients with FPF, emphasizing the need for improved strategies to recognize and manage hemorrhagic complications in this vulnerable population.

## Conclusion

This nationwide study demonstrated that 5.9% of elderly patients with FPF required TAE, and that the need for TAE was independently associated with worse clinical outcomes. We identified several clinical and physiological predictors of MAB, providing actionable insights to support early recognition and timely intervention in high-risk patients. Although FPF typically results from low-energy trauma, it can nonetheless lead to life-threatening hemorrhage. Recognizing these risk factors may help clinicians anticipate deterioration and inform clinical decision-making in this vulnerable population, such as through early CT angiography, activation of trauma teams, and preparation for massive transfusion protocols in selected high-risk cases. Future prospective studies are warranted to validate these findings and inform the development of evidence-based guidelines tailored to this increasingly prevalent and vulnerable patient population.

## Supplementary Information

Below is the link to the electronic supplementary material.

Supplementary Material 1

Supplementary Material 2

## Data Availability

The datasets generated and/or analysed during the current study are not publicly available because they are owned by the Japan Trauma Data Bank (JTDB) but are available from the corresponding author on reasonable request and with permission from JTDB.

## Declarations

### Online resources

Additional data are available in the supplementary materials (Online Resources 1–7).

## References

[1] Burge, R. et al. Incidence and economic burden of Osteoporosis-Related fractures in the united States, 2005–2025*. *J. Bone Min. Res.* **22**, 465–475 (2009). 10.1359/jbmr.061113 17144789

[2] Kannus, P., Parkkari, J., Niemi, S. & Sievänen, H. Low-Trauma pelvic fractures in elderly Finns in 1970–2013. *Calcif Tissue Int.* **97**, 577–580 (2015). 26319676 10.1007/s00223-015-0056-8

[3] Ramser, M. et al. The impact of specific fracture characteristics of low-energy fractures of the pelvis on mortality. *BMC Geriatr.* **22**, 669 (2022). 35971065 10.1186/s12877-022-03223-z PMC9377136

[4] Reito, A. et al. Mortality and comorbidity after non-operatively managed, low-energy pelvic fracture in patients over age 70: a comparison with an age-matched femoral neck fracture cohort and general population. *BMC Geriatr.* **19**, 315 (2019). 31744463 10.1186/s12877-019-1320-y PMC6862845

[5] Dietz, S. O., Hofmann, A. & Rommens, P. M. Haemorrhage in fragility fractures of the pelvis. *Eur. J. Trauma. Emerg. Surg.* **41**, 363–367 (2015). 26037987 10.1007/s00068-014-0452-1

[6] Weber, C. D. et al. Management of Life-Threatening arterial hemorrhage following a fragility fracture of the pelvis in the anticoagulated patient: case report and review of the literature. *Geriatr. Orthop. Surg. Rehabil*. **7**, 163–167 (2016). 27551576 10.1177/2151458516649642 PMC4976735

[7] Krappinger, D. et al. Hemorrhage after low-energy pelvic trauma. *J. Trauma. Acute Care Surg.* **72**, 437–442 (2012). 22439207 10.1097/ta.0b013e31822ad41f

[8] Coccolini, F. et al. Pelvic trauma: WSES classification and guidelines. *World J. Emerg. Surg.* **12**, 5 (2017). 28115984 10.1186/s13017-017-0117-6 PMC5241998

[9] Matsumoto, S. et al. Effectiveness and usage trends of hemorrhage control interventions in patients with pelvic fracture in shock. *World J. Surg.* **44**, 2229–2236 (2020). 32112165 10.1007/s00268-020-05441-1

[10] Cullinane, D. C. et al. Eastern association for the surgery of trauma practice management guidelines for hemorrhage in pelvic fracture–update and systematic review. *J. Trauma.* **71**, 1850–1868 (2011). 22182895 10.1097/TA.0b013e31823dca9a

[11] Maier, G. S. et al. Risk factors for pelvic insufficiency fractures and outcome after Conservative therapy. *Arch. Gerontol. Geriatr.* **67**, 80–85 (2016). 27448040 10.1016/j.archger.2016.06.020

[12] de Herdt, C. L. et al. Clinically relevant bleeding risk in low-energy fragility fractures of the pelvis in elderly patients. *Eur. J. Trauma. Emerg. Surg.* **50**, 1585–1589 (2024). 38478055 10.1007/s00068-024-02492-0

[13] Forssten, M. P. et al. Adverse outcomes following pelvic fracture: the critical role of frailty. *Eur. J. Trauma. Emerg. Surg.* **49**, 2623–2631 (2023). 37644193 10.1007/s00068-023-02355-0 PMC10728265

[14] Yoon, S. H. et al. Influence of comorbidities on functional outcomes in patients with surgically treated fragility hip fractures: a retrospective cohort study. *BMC Geriatr.* **21**, 283 (2021). 33910513 10.1186/s12877-021-02227-5 PMC8082882

[15] Vaidya, R. et al. Patients with pelvic fractures from blunt trauma. What is the cause of mortality and when? *Am. J. Surg.* **211**, 495–500 (2016). 26781723 10.1016/j.amjsurg.2015.08.038

