# Supplementary material for: Prevalence and risk factors for major arterial bleeding in fragility pelvic fractures in the aging population
Source: Sci Rep. 2025 Oct 31;15:38257. doi: 10.1038/s41598-025-22076-1 (PMC12578900; doi:10.1038/s41598-025-22076-1)
Supplement: Supplementary file 1 — Supplementary Material 1 [file 41598_2025_22076_MOESM1_ESM.pdf]

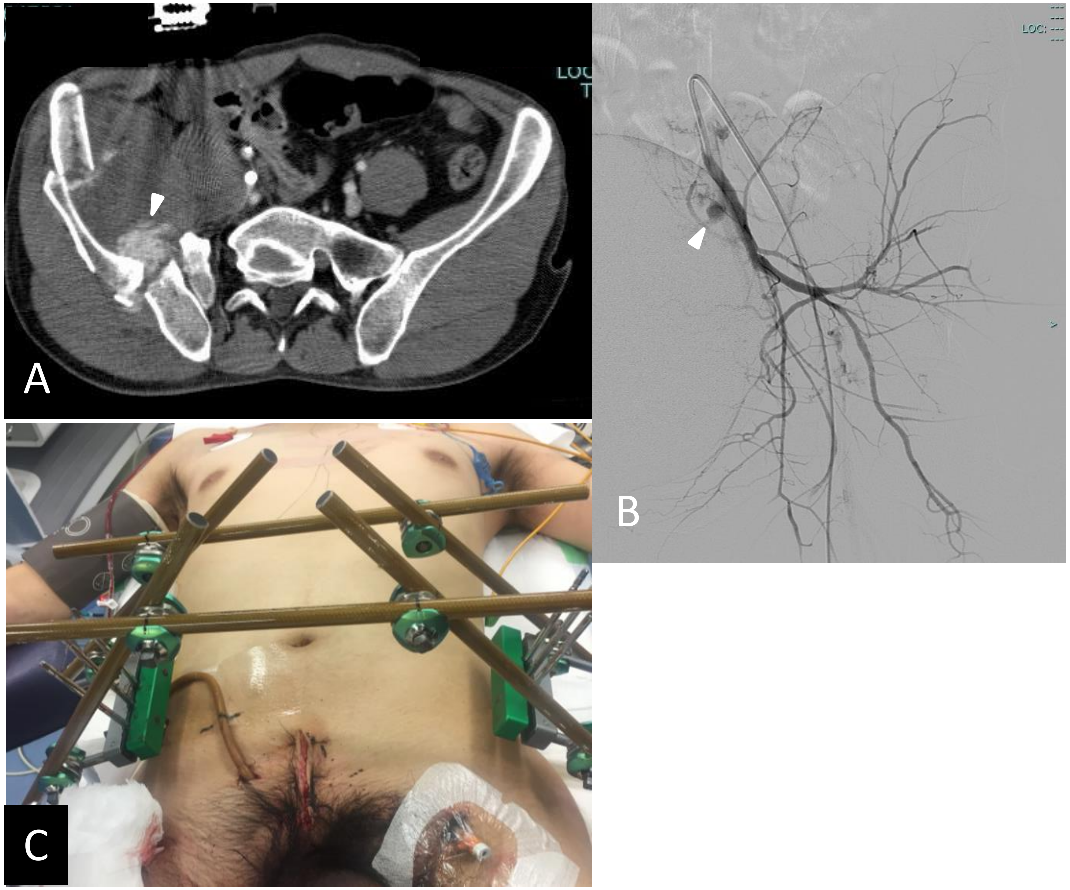

Supplementary information 1. Imaging and interventions for hemorrhage control in fragility pelvic fracture.
A: Contrast-enhanced computed tomography showing active extravasation in the pelvis (white arrow).
B: Angiography demonstrating contrast extravasation from a pelvic artery (white arrow).
C: Application of external fixation for pelvic stabilization.
