# Supplementary material for: Prevalence and risk factors for major arterial bleeding in fragility pelvic fractures in the aging population
Source: Sci Rep. 2025 Oct 31;15:38257. doi: 10.1038/s41598-025-22076-1 (PMC12578900; doi:10.1038/s41598-025-22076-1)
Supplement: Supplementary file 2 — Supplementary Material 2 [file 41598_2025_22076_MOESM2_ESM.docx]

| **Injury Description** | **AIS2008** | **AIS98** |
| --- | --- | --- |
| **Pelvic ring fracture** | 856100.2  856101.3  856151.2  856152.3  856161.3  856162.4  856163.4  856164.5  856171.4  856172.4  856173.5  856174.5 | 852600.2  852604.3  852600.2  852604.3  852604.3  852604.3  852608.4  852610.5  852606.4  852608.4  852610.5 |
| Acetabular fracture | 856200.2  856202.3  856251.2  856252.3  856261.2  856262.3  856271.2  856272.3 | 852600.2  852604.3  852600.2  852604.3  852600.2  852604.3  852600.2  852604.3 |

**Supplementary information 2. ICD-based AIS codes used to identify pelvic ring and acetabular fractures in the JTDB. Both AIS98 and AIS2008 codes are shown.**

**Supplementary information 3. Baseline Characteristics of Elderly Patients With Pelvic Fracture Due to Ground-Level Falls, Stratified by Presence of Major Arterial Bleeding Excluding Abdominal AIS>=3 (n=1,330)**

| Demographic Characteristics | Non-MAB (n=1260) | MAB (n=70) | Total (n=1330) | P-value |
| --- | --- | --- | --- | --- |
| Male sex, n (%) | 307 (24.4) | 30 (42.9) | 337 (25.3) | 0.001 |
| Age, median | 85 (79-89) | 86 (83-90) | 85 (79-90) | 0.085 |
| Age group (y), n (%) |  |  |  |  |
| 65-69 | 71 (5.6) | 1 (1.4) | 72 (5.4) | 0.151 |
| 70-74 | 116 (9.2) | 5 (7.1) | 121 (9.1) |  |
| 75-79 | 153 (12.1) | 4 (5.7) | 157 (11.8) |  |
| 80-84 | 244 (19.4) | 13 (18.6) | 257 (19.3) |  |
| 85-89 | 362 (28.7) | 27 (38.6) | 389 (29.2) |  |
| 90-94 | 218 (17.3) | 11 (15.7) | 229 (17.2) |  |
| 95+ | 96 (7.6) | 9 (12.9) | 105 (7.9) |  |
| Vital signs on hospital arrival |  |  |  |  |
| SBP, mm Hg | 142 (122-162) | 117 (95-143) | 141 (120-161) | <0.001 |
| SBP<90 mmHg, n (%) | 59 (4.7) | 16 (22.9) | 75 (5.6) | <0.001 |
| HR, bpm | 82 (72-94) | 81 (72-95) | 82 (72-94) | 0.822 |
| HR>120 bpm, n (%) | 36 (2.9) | 4 (5.7) | 40 (3.0) | 0.155 |
| Temperature, °C | 36 (36-37) | 36 (35-36) | 36 (36-37) | <0.001 |
| GCS score | 15 (14-15) | 15 (13-15) | 15 (14-15) | <0.001 |
| GCS<9, n (%) | 35 (2.8) | 8 (11.4%) | 43 (3.2) | <0.001 |
| ISS | 9 (4-13) | 16 (14-25) | 9 (4-13) | <0.001 |
| Severe injured region, AIS score |  |  |  |  |
| Head AIS≥3, n (%) | 91 (7.2) | 6 (8.6) | 97 (7.3) | 0.852 |
| Chest AIS≥3, n (%) | 86 (6.8) | 5 (7.1) | 91 (6.8) | 1.000 |
| Abdomen AIS≥3, n (%) | 0 (0) | 0 (0) | 0 (0) | 1.000 |
| Upper extremity AIS≥2, n (%) | 147 (11.7) | 5 (7.1) | 152 (11.4) | 0.335 |
| Lower extremity AIS≥3, n (%) | 525 (41.7) | 58 (82.9) | 583 (43.8) | <0.001 |
| Femur fracture, n (%) | 122 (9.7) | 5 (7.1) | 127 (9.5) | 0.621 |

Continuous variables were presented as the mean ± SD or the median (IQR). Categorical variables were presented as number (%).

SBP: Systolic Blood Pressure, HR: Heart Rate, GCS: Glasgow Coma Scale, ISS: Injury Severity Score, AIS: Abbreviated Injury Scale

**Supplementary information 4. Multivariable Logistic Regression Identifying Predictors of Major Arterial Bleeding in Elderly Patients with Fragility Pelvic Fractures Excluding Abdominal AIS>=3 (n=1,330)**

| **Major arterial bleeding** | **Odds ratio** | **95% CI** | | **P-value** |
| --- | --- | --- | --- | --- |
| **Age** | 1.04 | 1.01 | 1.07 | <0.001 |
| **Male** | 2.54 | 1.52 | 4.25 | <0.001 |
| **Cerebral vascular disease** | 2.07 | 1.14 | 3.74 | 0.016 |
| **Liver disease** | 4.64 | 1.49 | 14.4 | 0.007 |
| **SBP<90 mmHg** | 5.90 | 3.12 | 11.20 | <0.001 |
|  |  |  |  |  |
| **Mortality** |  |  |  |  |
| **Male** | 2.04 | 1.12 | 3.71 | 0.019 |
| **GCS<9** | 9.41 | 4.29 | 20.60 | <0.001 |
| **MAB** | 5.28 | 2.52 | 11.10 | <0.001 |
|  |  |  |  |  |

OR: Odds Ratio, CI: Confidence Interval, SBP: Systolic Blood Pressure, GCS: Glasgow Coma Scale, MAB: Major Arterial Bleeding

**Supplementary information 5. Baseline Characteristics of Elderly Patients With Pelvic Fracture Due to Ground-Level Falls, Stratified by Presence of Major Arterial Bleeding Excluding Head, Chest, Abdominal AIS>=3 (n=1,217)**

| Demographic Characteristics | Non-MAB (n=1155) | MAB (n=62) | Total (n=1217) | P-value |
| --- | --- | --- | --- | --- |
| Male sex, n (%) | 265 (22.9) | 23 (37.1) | 288 (23.7) | 0.016 |
| Age, median | 85 (79-90) | 86 (83-90) | 85 (79-90) | 0.111 |
| Age group (y), n (%) |  |  |  |  |
| 65-69 | 65 (5.6) | 1 (1.6) | 66 (5.4) | **0.139** |
| 70-74 | 104 (9.0) | 5 (8.1) | 109 (9.0) |  |
| 75-79 | 137 (11.9) | 4 (6.5) | 141 (11.6) |  |
| 80-84 | 224 (19.4) | 9 (14.5) | 233 (19.1) |  |
| 85-89 | 334 (28.9) | 25 (40.3) | 359 (29.5) |  |
| 90-94 | 197 (17.1) | 9 (14.5) | 206 (16.9) |  |
| 95+ | 94 (8.1) | 9 (14.5) | 103 (8.5) |  |
| Vital signs on hospital arrival |  |  |  |  |
| SBP, mm Hg | 143 (122-162) | 115 (93-143) | 142 (121-162) | <0.001 |
| SBP<90 mmHg, n (%) | 48 (4.2) | 15 (24.2) | 63 (5.2) | <0.001 |
| HR, bpm | 82 (72-94) | 80 (72-93) | 82 (72-94) | 0.772 |
| HR>120 bpm, n (%) | 32 (2.8) | 3 (4.8) | 35 (2.9) | 0.418 |
| Temperature, °C | 36 (36-37) | 36 (35-36) | 36 (36-37) | <0.001 |
| GCS score | 15 (14-15) | 15 (14-15) | 15 (14-15) | 0.001 |
| GCS<9, n (%) | 18 (1.6) | 5 (8.1) | 23 (1.9) | 0.001 |
| ISS | 9 (4-9) | 16 (13-25) | 9 (4-10) | 0.000 |
| Severe injured region, AIS score |  |  |  |  |
| Head AIS≥3, n (%) | 0 (0) | 0 (0) | 0 (0) | 1.000 |
| Chest AIS≥3, n (%) | 0 (0) | 0 (0) | 0 (0) | 1.000 |
| Abdomen AIS≥3, n (%) | 0 (0) | 0 (0) | 0 (0) | 1.000 |
| Upper extremity AIS≥2, n (%) | 124 (10.7) | 4 (6.5) | 128 (10.5) | 0.394 |
| Lower extremity AIS≥3, n (%) | 492 (42.6) | 53 (85.5) | 545 (44.8) | <0.001 |
| Femur fracture, n (%) | 113 (9.8) | 4 (6.5) | 117 (9.6) | 0.509 |

Continuous variables were presented as the mean ± SD or the median (IQR). Categorical variables were presented as number (%).

SBP: Systolic Blood Pressure, HR: Heart Rate, GCS: Glasgow Coma Scale, ISS: Injury Severity Score, AIS: Abbreviated Injury Scale

**Supplementary information 6. Multivariable Logistic Regression Identifying Predictors of Major Arterial Bleeding in Elderly Patients with Fragility Pelvic Fractures Excluding Head, Chest, Abdominal AIS>=3 (n=1,217)**

| **Major arterial bleeding** | **Odds ratio** | **95% CI** | | **P-value** |
| --- | --- | --- | --- | --- |
| **Age** | 1.04 | 1.00 | 1.08 | 0.034 |
| **Male** | 2.30 | 1.31 | 4.03 | 0.004 |
| **Cerebral vascular disease** | 2.12 | 1.12 | 3.99 | <0.001 |
| **Liver disease** | 4.68 | 1.49 | 14.7 | 0.008 |
| **SBP<90 mmHg** | 7.66 | 3.90 | 15.00 | <0.001 |
|  |  |  |  |  |
| **Mortality** |  |  |  |  |
| **Male** | 3.20 | 1.64 | 6.25 | <0.001 |
| **GCS<9** | 10.90 | 3.74 | 31.80 | <0.001 |
| **MAB** | 4.54 | 1.95 | 10.60 | <0.001 |
|  |  |  |  |  |

OR: Odds Ratio, CI: Confidence Interval, SBP: Systolic Blood Pressure, GCS: Glasgow Coma Scale, MAB: Major Arterial Bleeding

**Supplementary information 7. Mortality distribution by time from admission in elderly patients with fragility pelvic fractures in the JTDB from 2010 to 2021.**

|  | n | % | Cumulative % |
| --- | --- | --- | --- |
| Died<24 hours | 7 | 13 | 13 |
| Died 24-48 hours | 8 | 15 | 28 |
| Died 48 hours – 7 days | 12 | 22 | 50 |
| Died 7 days< | 27 | 50 | 100 |

JTDB: Japan Trauma Data Bank, CT: computed tomography, REBOA; Resuscitative Endovascular Balloon Occlusion of the Aorta, LOS: length of stay.
